# Temporal dynamics of hot desert microbial communities reveal structural and functional responses to water input

**DOI:** 10.1038/srep34434

**Published:** 2016-09-29

**Authors:** Alacia Armstrong, Angel Valverde, Jean-Baptiste Ramond, Thulani P. Makhalanyane, Janet K. Jansson, David W. Hopkins, Thomas J. Aspray, Mary Seely, Marla I. Trindade, Don A. Cowan

**Affiliations:** 1 Centre for Microbial Ecology and Genomics (CMEG), Genomics Research Institute, Department of Genetics, University of Pretoria, Pretoria 0002, South Africa; 2 Earth and Biological Sciences Directorate, Pacific Northwest National Laboratory, Richland, WA 99352, USA; 3 The Royal Agricultural University, Cirencester, Gloucestershire GL7 6JS, UK; 4 School of Life Sciences, Heriot-Watt University, Edinburgh, EH14 4AS, UK; 5 Gobabeb Training and Research Centre (GTRC), Walvis Bay, Namibia; 6 Animal, Plant and Environmental Sciences, University of Witwatersrand, Johannesburg, South Africa; 7 Institute for Microbial Biotechnology and Metagenomics (IMBM). University of the Western Cape, Bellville 7535, South Africa

## Abstract

The temporal dynamics of desert soil microbial communities are poorly understood. Given the implications for ecosystem functioning under a global change scenario, a better understanding of desert microbial community stability is crucial. Here, we sampled soils in the central Namib Desert on sixteen different occasions over a one-year period. Using Illumina-based amplicon sequencing of the 16S rRNA gene, we found that α-diversity (richness) was more variable at a given sampling date (spatial variability) than over the course of one year (temporal variability). Community composition remained essentially unchanged across the first 10 months, indicating that spatial sampling might be more important than temporal sampling when assessing β-diversity patterns in desert soils. However, a major shift in microbial community composition was found following a single precipitation event. This shift in composition was associated with a rapid increase in CO_2_ respiration and productivity, supporting the view that desert soil microbial communities respond rapidly to re-wetting and that this response may be the result of both taxon-specific selection and changes in the availability or accessibility of organic substrates. Recovery to quasi pre-disturbance community composition was achieved within one month after rainfall.

A major goal in microbial community ecology is to understand the factors that underlie observed patterns in species distribution. As such, investigating changes in microbial community composition and how these changes relate to physical distance among communities has been the focus of considerable research (e.g. refs 1, 2, 3). Spatial changes in microbial community composition have been linked to four major processes: selection, drift, dispersal and mutation^4^.

In addition to varying in space, microbial communities are also dynamic over time^5^. In soils, studies have shown that community composition can change across days^6^, seasons^7^ and years^8^, as a result of both biotic and abiotic processes. However, most investigations on the temporal variability of soil microbial communities have focused on Northern hemisphere temperate environments^7^,^9^, where such soils are commonly characterised by high primary productivity, a major driver for compositional variation^10^. Clearly, more ecologically diverse communities should be studied in order to better understand temporal patterns in microbial diversity.

Deserts constitute the largest biome on Earth, covering around 20% of the global land surface^11^. Hot desert ecosystems are characterized by scant, erratic and low precipitation inputs, high temperatures with extreme seasonal and diurnal fluctuations in temperature, low nutrient status, high levels of incident ultraviolet (UV) radiation and physical disturbance^12^. However, despite these environmental constraints, deserts are globally significant, as they sustain *ca*. 6% of the human population, host many endemic plants and animals and store almost one third of total terrestrial carbon^13^,^14^. Nevertheless, the temporal variation of microbial communities has seldom been investigated in deserts^3^,^15^,^16^.

Combining Illumina MiSeq amplicon sequencing of replicated surface-soil samples, chlorophyll *a* (chl*a*) content, CO_2_ respiration rates and environmental (climate and soil chemistry) data, we investigated the temporal variability of bacterial and archaeal communities, and their associated activities and productivities, over a one-year period in the Namib Desert gravel plains. Because deserts are low nutrient and physically stressful environments with comparatively small species pool sizes^17^, we hypothesize that the rate of change in α-diversity over time should be lower relative to more benign edaphic ecosystems. We also expect a low β-diversity turnover, which will be accompanied by small changes in activity and productivity. Whether significant temporal shifts in either α- or β-diversity do occur, we hypothesize that these shifts should be linked to changes in soil conditions.

## Results and Discussion

We investigated the temporal variability of edaphic microbial communities (bacteria and archaea) in the Namib Desert gravel plains over a one-year period. Macro- and micro-environmental conditions, and soil nutrient status data were collected throughout the period. We also linked this information to shifts in microbial activity (CO_2_ respiration rates) and productivity (chl*a* content).

**Figure 1:**
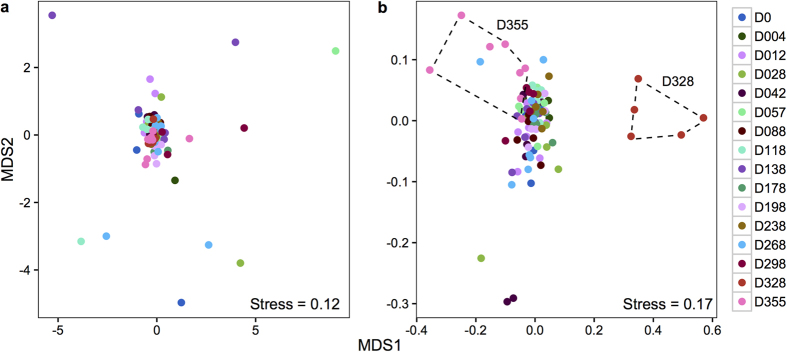
Non-metric multidimensional scaling ordination plots. (**a**) Soil chemistry profiles (Euclidean distances with standardized data), excluding moisture content. To appreciate the changes induced by the addition of soil moisture content in the grouping of the samples see Supp. Figure 1. (**b**) Sequencing data (Bray-Curtis distances after Hellinger transformation). D328, microbial communities 3 days after rainfall; D355, microbial communities 30 days after rainfall. Samples that are closer together are more similar in soil chemistry or microbial community composition.

Soil chemical analysis results showed low nutrient levels (Supp. Table 1), comparable to those previously found in Namib Desert soils^18^,^19^,^20^,^21^. Soil chemical properties remained stable throughout the year (Fig. 1a), with no significant differences in any of the parameters measured (excluding soil moisture content) according to sampling date or season. Air temperature and relative humidity showed a similar pattern (data not shown). Rainfall during the sampling period was restricted to a single precipitation event (38 mm) on day 325. Rainfall in the Namib Desert is typically highly stochastic, both on temporal and spatial scales^22^. As expected, the soil moisture contents responded to the single precipitation event by a 100-fold increase between day 298 (30 days pre-rainfall) and day 328 (three days post-rainfall) (Supp. Table 1, Supp. Figure 1).

Respiration rates were low for most of the year (mean 0.022 mg CO_2_-C g^−1^ soil hour^−1^; SE = 0.001), but were *ca.* 4-fold higher 3 days after rainfall (mean 0.079 mg CO_2_-C g^−1^ soil hour^−1^; SE  = 0.015) and remained well above average on day 355 (mean 0.065 mg CO_2_-C g^−1^ soil hour^−1^; SE  =  0.007), 30 days after the rainfall event. Rainfall induced CO_2_ pulses and increases in other microbial processes, resulting from resurrection of microbial communities and changing accessibility to organic substrates, have been shown to occur in Mediterranean^23^,^24^ and desert soil environments^25^, both of which have long periods of drought interspaced with precipitation events. The cause of this phenomenon, which has become known as the “Birch effect” (see ref. 23 and references therein), is not completely understood, but the likely contributory biophysical conditions are: (1) drying and rewetting shatters soil aggregates and exposes previously unavailable organic substrates for decomposition; (2) microorganisms killed by soil drying are decomposed on rewetting to release their nutrients; (3) there is a spontaneous rapid increase in microbial biomass in response to the availability of water perhaps associated with the breaking of dormancy; and (4) there is a microbial hypo-osmotic stress response. These mechanisms are not mutually exclusive^26^. Irrespective of the mechanisms and whether they operate simultaneously, with the projected intensification of intra-annual precipitation in desert systems^27^, our results suggest that these environments may become increasing sources of net CO_2_ output, potentially having a positive (and possibly detrimental) feedback on atmospheric CO_2_-linked global change processes.

Chl*a* content concentrations (a proxy for productivity) were positively correlated with respiration rates (Spearman p = 0.5, P < 0.05). The average chl*a* concentration in pre-rainfall communities was 0.13 mg chl*a* mg^-^¹ soil, but 1.96 mg chl*a* mg^−^¹ soil for post-rainfall communities. Positive relationships between microbial activity, productivity and moisture content have been shown across different soil types^28^. The increase in soil moisture is probably altering conditions for desert soil microbial communities, mobilizing nutrients and salts, and stimulating primary productivity by cyanobacterial populations^13^. Indeed, cyanobacteria have been reported to require minimum soil moisture level of 15% to be photosynthetically active^29^.

Amplicon sequencing analysis of the 102 samples collected over 12 months yielded a total of 2170 OTUs (97% identity cut-off) from 33456 sequences. The number of OTUs per sample (α-diversity) ranged from 125 to 208, values similar to those reported for Atacama Desert soils^30^. We do not expect this variability to be related to sequencing depth, as all samples were rarefied to the same number of reads (328). While we do not have a complete justification for the relatively low number of reads, similar results have been obtained in other nutrient-limited environments^31^,^32^,^33^. Additionally, one focus of the study is on β-diversity, and it has been shown that shallow sequencing captures similar β-diversity patterns compared to deep sequencing^34^. This is because β-diversity is less affected by the number of samples reads, as in the case of α-diversity.

**Figure 2:**
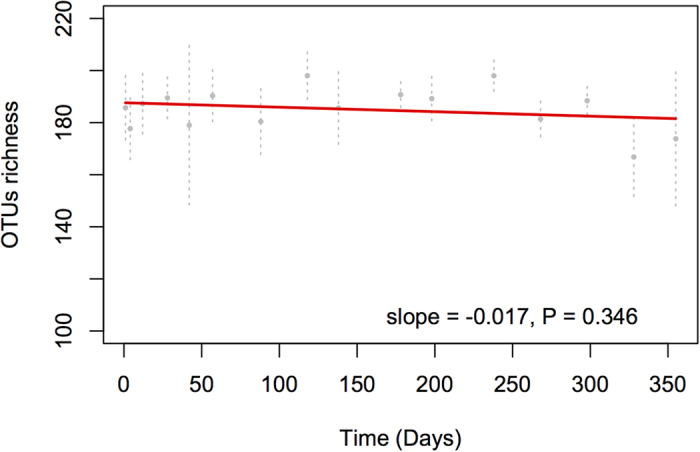
Global trend analysis in α-diversity (richness). A linear trend is fit (red line) using generalized least squares (GLS) applying a model with AR1 temporal autocorrelation of errors. The average value on each sampling date is plotted with ± S.D. of the mean.

No significant correlations between α-diversity and any of the environmental variables were observed (not shown). A possible explanation for this observation is that α-diversity in these soils may be driven by unmeasured biotic and abiotic variables. As hypothesized, despite some differences between samples collected at a given time, the temporal variability of α-diversity was very low (Fig. 2), indicating that spatial variability in richness may exceed temporal variability in desert soil microbial communities. This is not an unexpected result, as soils are typically spatially heterogeneous, but is not in agreement with results of temporal studies performed in other terrestrial^9^ and aquatic^35^ ecosystems. The contrast in α-diversity patterns between this and previous studies is probably related primarily to the aridity, because in the absence of water, transport of microorganisms and nutrients will be limited.

The slope value (0.39) of the species-time relationship, an index of temporal turnover, was within the lower range (0.24–0.61) found for microbial communities in a meta-analysis^5^. This suggests that the taxa present at this location do not change appreciably over time, in accordance with previous results^36^. This low turnover could be a product of slow growth rates overall, the low degree of variability in soil properties, low regional species pool size, or perhaps because of the high degree of dormancy in these microbial communities^37^.

**Figure 3:**
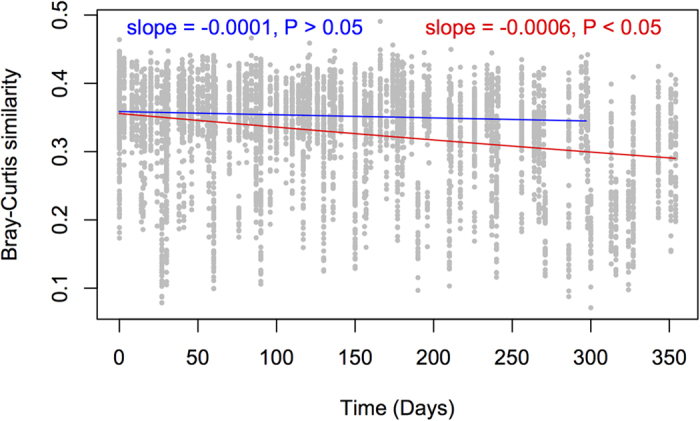
Temporal decay curves for microbial communities. The blue line denotes the regression for communities sampled before the rainfall event. The red line denotes a separate regression for all sampling points. Both lines were obtained using the generalized dissimilarity model of Millar *et al.*^58^. Only the slope of the solid red line, including the samples after the rainfall event, is significantly less than zero.

As for α-diversity, β-diversity values (Bray-Curtis dissimilarities *ca*. 0.63) did not change significantly over time (Supp. Figure 2), even after the rainfall event and the consequent increase in soil moisture and productivity. An increase in β-diversity with rising moisture^38^ and productivity^10^ is frequently observed in microbial communities. A possible reason for the absence of a similar pattern in these communities might be that rainfall acted as a selection factor affecting more members of the community, which may result in lower β-diversity values^39^. Furthermore, the insignificant time-decay relationship confirmed that the microbial communities remained largely unchanged for 10 of the 12 months (Fig. 3), from the beginning of the study until the last sampling point before the rainfall event. Microbial community shifts commonly result from changes in environmental variables^40^, which remained relatively constant in the Namib Desert during 10 months of the 12-month sampling period. Altogether, these results suggest that environmental filtering, as a consequence of low water content and nutrient concentrations, is critical in shaping these microbial communities.

The dataset indicated that the single rainfall event greatly affected the soil microbial communities. A nonmetric multidimensional scaling plot showed differences in community structure between pre- and post-rainfall samples (Fig. 1b). PERMANOVA: P < 0.05 in all pairwise comparison including D328), which resulted in a significant decrease in the microbial community similarity with time over the full year (Fig. 3). This is in line with a recent study that showed that microbial communities from the Namib Desert responded rapidly to (intense) water inputs^18^. Furthermore, post-rainfall samples formed two separated clusters (Fig. 1b). The first cluster included samples obtained 3 days after the precipitation event (day 328), whereas the second corresponded to those collected 30 days after the precipitation event (day 355). Interestingly, the latter communities (day 355) were more similar to those from pre-rainfall samples than to those from 3 days post-rainfall (D328), suggesting that the microbial community structure tended to revert to the pre-disturbance status, a sign of microbial community resilience^41^. The unique clustering of the microbial communities 3 days after the precipitation event was driven both by the detection of 137 new taxa (Supp. Figure 3) and by changes in the relative abundance of taxa previously identified (see below). Many rare taxa that are below the detection limit can become detectable when dominant species are reduced in their relative abundance. Alternatively, this high short-term community turnover may be a consequence of stochastic dispersal from the surrounding environment^42^.

**Figure 4:**
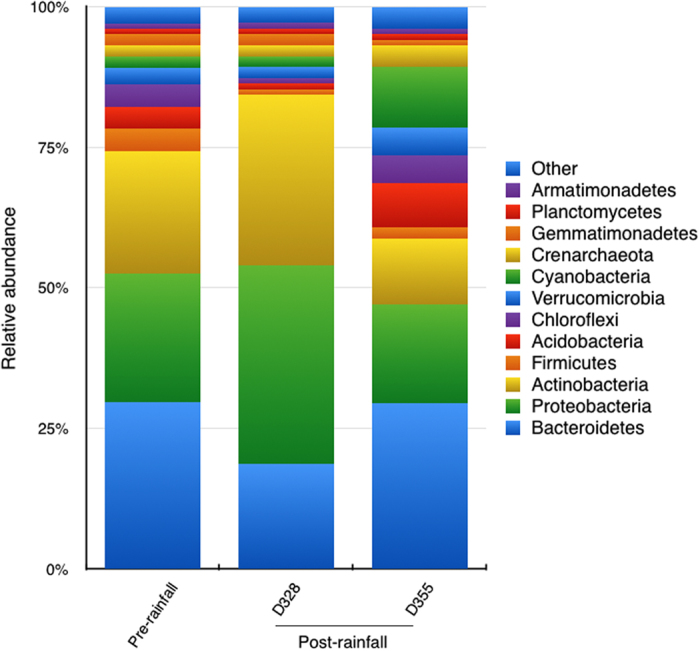
Taxonomic distribution, phylum level, of bacterial OTUs (97% cutoff). Affiliation was performed using the Ribosomal Database Project Classifier with a confidence threshold of 80%. Pre-rainfall, microbial communities before the rainfall event; D328, microbial communities 3 days after rainfall; D355, microbial communities 30 days after rainfall.

A total of 19 bacterial phyla and 2 archaeal phyla were identified in all samples. Microbial communities were dominated by *Bacteroidetes* (29% of all sequences), *Proteobacteria* (23%, mostly Alphaproteobacteria (17%) and Betaproteobacteria (3%)) and *Actinobacteria* (22%); followed by Firmicutes (4%), Acidobacteria (4%), Chloroflexi (4%) and Verrucomicrobia (3%). Archaea (mainly phylum *Crenarchaeota*) contributed 2% of the sequences. Similar results have been found in other studies^17^,^43^,^44^, indicating that common members of these phyla are probably well adapted to survive and possibly thrive in desert soils^20^. The relative abundance of *Proteobacteria* (mainly *Betaproteobacteria*, Supp. Figure 4) and *Actinobacteria* increased after the single rainfall event (Fig. 4). Previous studies have also observed that *Actinobacteria*^9^ and *Betaproteobacteria*^45^ tend to dominate with increasing moisture. This may reflect the nutritional preferences of these two groups, as members of the *Actinobacteria* and *Betaproteobacteria* seem to respond positively to carbon availability^46^, which is thought to increase with soil moisture^47^. Nevertheless *Bacteroidetes*, also proposed as a copiotrophic group^46^, showed the opposite trend in our data, decreasing with moisture (Fig. 4). *Bacteroidetes* were also shown to decrease in biocrust communities following a simulated rainfall event^43^. Overall, these apparently contradictory results can be explained in light of recent investigations showing that physiological traits and not phylogeny are better predictors of moisture preferences^48^.

In summary, we have demonstrated low temporal variability over a year period in desert soil microbial communities. Although a major shift in microbial community composition was induced by a precipitation event. The precipitation-induced shifts in microbial community composition increased CO_2_ respiration and productivity, demonstrating that desert soil microbial communities can respond to water addition. The major community shift after wetting accompanied by the occurrence of increased soil respiration rates and chl*a* concentrations is consistent with a multi-mechanisms response, involving both taxon-specific selection and changes in the availability or accessibility of organic substrates. In general, the variability in microbial communities within the sampling location (spatial variability) at a given time was higher than the intra-annual temporal variability, suggesting that sampling more sites (preferably at different locations) might be more important than frequent sampling if the aim is to describe compositional changes in desert soil microbial communities and their implications for ecosystem functioning. Nevertheless, collecting time series of soil microbial communities is important to further understanding community stability, given ongoing global climate change disturbances.

## Materials and Methods

### Study site description and sampling strategy

Soil samples were collected between 08:00 and 10:00 (am) from the gravel plains in the central Namib Desert (S23° 33.302', E15° 3.288) near Gobabeb over a 12-month period (from 04 May 2012 to 28 April 2013). The study area (8100 m^2^) consisted of 81 (10 × 10 m) plots on a south-facing 2º slope.

Using a random number generator, eight plots were sampled during each of the 16 sampling campaigns (days 0, 4, 12, 28, 42, 57, 88, 118, 138, 178, 198, 238, 268, 298, 328, 355). From each plot, surface soil samples (0–10 cm) were collected using a 1-m^2^ grid divided into 16 quadrats. These samples were pooled and homogenized to obtain a single sample. Soil samples were maintained at −20 °C in sterile Whirl-Pak bags (Nasco International, Fort Atkinson, WI, USA). A total of 128 samples were collected (16 sampling campaigns each consisting of 8 samples).

### Macroenvironmental variables

Air temperature and relative humidity data were obtained from the Gobabeb Land Surface Temperature (LST) weather station using thermal infrared satellite measurements. The station was established by the Karlsruhe Institute of Technology^7^ in the central Namib Desert gravel plains (23°33′S, 15°03′E), located 400 m above sea level (350 m west of the study site). The station instruments were mounted at 2 m, measuring air temperature and relative humidity at 1-minute intervals. Rainfall data was obtained from the Gobabeb Research and Training Centre located 1.5 km east of the study area (http://www.gobabebtrc.org/).

### Soil physicochemical characterisation

Analysis of soil samples was conducted at the Soil Science Laboratory of the University of Pretoria, South Africa in accordance with the standard procedures (SSSA, 1996). Prior to analysis, soil samples were sieved (2 mm) and dried overnight at 50 °C.

The slurry technique was used to measure pH (1:3 soil/deionised water) with a Crison Bench pH meter (Crison Instruments, Barcelona, Spain) after allowing soil to settle for 30 min. Soil N-NO_3_^-^, N-NH_4_^+^ and cation exchange capacity (CEC) were determined by extraction (2M KCl) with subsequent titration. Total P was measured using the P Bray method and total C was measured using the Walkley-Black method. Ammonium acetate extraction was used to measure salt concentrations (K^+^, Ca^2+^, Mg^2+^, Na^+^), analysed using inductively coupled plasma atomic emission spectroscopy (ICP-OES; Spectro Genesis, Spectro Analytical Instruments GmbH, Germany). Moisture content was determined by oven drying 10 g of soil at 60 °C for 48 h and comparing the weight of soil pre- and post-drying.

### Soil respiration (decomposition)

Between 8.5 and 10.5 g soil were incubated for up to 10 days in a conductiometric respirometer (Nordgren, 1988) at 20˚C in the dark, and CO_2_ production measured at hourly intervals. CO_2_ production data were used to estimate soil respiration rate by linear regression of the CO_2_ over the first 2–3 days. Linear regression was significant (P < 0.05) in all cases. The mean and standard errors were calculated for all replicates after the replicates that gave the largest and smallest estimates had been removed. In no case was the mean estimate dependent on fewer than three replicates. The extreme replicates were removed because observable fragments of organic matter or salt encrustations led to anomalously high or unreproducibly low respiration rates, which in neither case produced statistically significant linear regressions of CO_2_ production with time.

### Chlorophyll a content (productivity)

Chl*a* extraction was performed on frozen (−20 °C) soil samples using a modification of the method described by Richie *et al.* Briefly, 3 g of soil was added to a sterile glass vial containing 8 ml 90% ethanol and incubated at 72 °C for 10 min and subsequently sonicated at 100% amplitude for 2 min. The supernatant was passed through a 0.2 mm filter into a glass test tube and the absorbance measured at 665 nm. Background fluorescence was determined by measuring absorbance at 750 nm. Chl*a* absorbance was calculated according to the formula


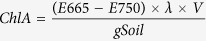


where: *E665* = Absorbance at 665 nm, *E750* = Absorbance at 750 nm, *V* = volume of extraction solution, *λ* = 11.9035 the cyanobacterial-specific coefficient and *gSoil* corresponds to the mass of soil^49^.

### DNA extraction and 16S rRNA gene amplicon barcoded sequencing

Metagenomic DNA was extracted from 0.25 g of soil using the PowerSoil^®^- htp 96-Well Soil DNA Isolation (MoBio, Carlsbad, CA, USA) following manufacturer’s instructions. In total, DNA was extracted from 128 samples according to the methods used by the Earth Microbiome Project (http://www.Earthmicrobiome.Org/emp-standard-protocols/dna-extraction-protocol/). Sample sequencing was performed using bacterial/archaeal 515f and 806r error-correcting barcoded primers as described previously^50^. Illumina MiSeq sequence data was screened using the QIIME toolkit^51^ with the following parameters: quality score >25, sequence length >200 and <400, maximum homopolymer of 6, 0 maximum ambiguous bases, and 0 mismatched bases in the primers and barcodes. Sequence counts before and after quality filtering were 348687 and 124597, respectively. Following chimera removal (USEARCH 6.1^52^), 115102 sequences remained. OTUs were picked at the 97% identity level using UCLUST^52^ with the default settings. Phylogenetic identification was performed against the most recent Greengenes database (gg_13_5)^53^. After removal of singletons, samples were rarefied to 328 reads, which represents the lowest number of sequences obtained for a single sample. In total 26 samples, which failed quality control, were excluded from further analysis, yielding a total of 102 samples.

### Data analysis

Biotic and abiotic data sets were analysed using the R environment for statistical computing (version 3.0.3^54^) using vegan^55^ and custom scripts. Differences in abiotic data were assessed using Wilcoxon-Mann-Whitney *post hoc* tests after ensuring that an overall Kruskal-Wallis test was significant (P < 0.05). Microbial community similarity was calculated using the Bray-Curtis index and visualised using non-metric multidimensional scaling (NMDS). Permutational multivariate analysis of variance (PERMANOVA) was used to test for statistically significant variance between groups^56^.

Richness trend analysis was performed using generalized least squares (GLS) with AR1 temporal autocorrelation of errors^57^. Distance-decay relationships between microbial community composition and temporal distance were plotted using the generalized dissimilarity model of Millar *et al.*^58^. Species-time relationship (STR) was constructed in R by calculating richness using the moving window approach of White *et al.*^59^. This approach was used to allow comparison with the results from microbial communities reported in shade *et al.*^5^. Analyses were performed on Hellinger transformed sequence data and on standardized environmental data.

## Additional Information

**Accession codes**: The sequence data generated in this study were deposited in the NCBI Sequence Read Archive and are available under the project number: SRP066886.

**How to cite this article**: Armstrong, A. *et al.* Temporal dynamics of hot desert microbial communities reveal structural and functional responses to water input. *Sci. Rep.* **6**, 34434; doi: 10.1038/srep34434 (2016).

## Supplementary Material

Supplementary Information
