# Supplementary material for: Temporal dynamics of hot desert microbial communities reveal structural and functional responses to water input
Source: Sci Rep. 2016 Sep 29;6:34434. doi: 10.1038/srep34434 (PMC5041089; doi:10.1038/srep34434)

Supplementary material for:

"Temporal dynamics of hot desert microbial communities reveal structural and functional responses to water input"

Alacia Armstrong, Angel Valverde, Jean-Baptiste Ramond, Thulani P. Makhalanyane, Janet Jansson, David W. Hopkins, Thomas J Aspray, Mary Seely, Marla I. Trindade and Don A. Cowan

Supp. Table 1. Soil chemical properties and moisture content. Mean ± Standard deviation (SD).

Supp. Figure 1. Non-metric multidimensional scaling ordination plots of soil chemistry profiles including moisture content. See difference with figure 1 in the main text. D328, microbial communities 3 days after rainfall.

Supp. Figure 2. Global trend analysis in beta-diversity (Bray-Curtis dissimilarity). A linear trend is fit using generalized least squares (GLS) applying a model with AR1 temporal autocorrelation of errors (red line). The average value on each sampling date is plotted with ± S.D. of the mean.

Supp. Figure 3. Venn diagram depicting the number of shared and unique OTUs (97% cut-off).

Supp. Figure 4. Bar chart showing the proportion of the different classes within the Proteobacteria for the pre- and post-rainfall samples.

Supp. Table 1.

Day	pH	Organic C (%)	P (μg g^{-1})	NH_4^+ (μg g^{-1})	NO_3^- (μg g^{-1})	CEC (cmol$^+$ kg^{-1})	Ca+ (μg g^{-1})	K+ (μg g^{-1})	Mg+ (μg g^{-1})	Na+ (μg g^{-1})	Moisture (%)
**D0**	8.7±0.7	0.1±0	1.2±0.2	2.5±0.4	1.1±0.3	9.2±3.0	1306.0±171	250.7±66	71.1±20	263.9±54	6.0±2
**D004**	8.9±0.2	0.1±0	1.3±0.1	2.2±0.5	0.8±0.2	7.7±3.3	1636.3±719	220.5±61	64.3±12	57.2±20	6.5±3
**D012**	8.7±0.4	0.1±0	1.0±0.4	2.5±0.3	1.6±0.5	4.9±2.5	1159.0±134	191.0±16	58.8±5	18.0±2	4.6±2
**D028**	8.7±0.6	0.1±0	1.1±0.3	2.3±0.3	1.6±0.2	2.2±0.9	1562.2±773	259.9±111	72.1±21	27.9±4	3.5±2
**D042**	8.7±0.3	0.1±0	1.0±0.1	2.4±0.6	0.8±0.3	3.2±1.1	1168.4±143	187.0±15	59.4±4	18.1±7	4.7±3
**D057**	8.8±0.1	0.1±0	1.1±0.3	2.3±0.2	2.8±0.4	5.6±2.6	1683.9±820	202.1±35	80.4±43	52.6±4	5.8±5
**D088**	8.8±0.1	0.1±0	1.1±0.1	2.0±0.2	0.8±0.4	4.8±1.6	1212.1±170	197.2±35	58.5±4	83.9±14	0.5±0.5
**D118**	8.7±0.2	0.1±0	1.2±0.2	3.0±0.1	0.8±0.2	5.1±2.4	1368.5±341	188.3±32	60.8±5	21.6±8	0.9±0.2
**D138**	8.8±0.5	0.1±0	1.3±0.1	2.2±0.4	2.7±0.4	4.7±1.7	1899.1±820	203.2±42	81.1±31	25.1±10	0.8±0.4
**D178**	8.8±0.5	0.1±0	1.4±0.1	2.3±0.5	1.8±0.4	5.2±1.3	1419.1±448	214.8±29	66.1±5	36.8±15	0.8±0.6
**D198**	7.8±0.4	0.1±0	1.4±0.1	2.8±0.5	2.7±0.4	5.6±1.4	1454.1±399	223.0±8	69.3±2	25.6±7	1.2±0.6
**D238**	8.9±0.4	0.1±0	1.3±0.1	3.0±0.1	2.3±0.8	7.1±1.4	1393.2±303	197.9±7	66.9±3	23.1±3	0.6±0.4
**D268**	8.8±0.8	0.1±0	1.3±0.2	2.8±0.3	2.8±0.9	5.9±1.3	1571.3±371	227.4±31	66.1±3	195.3±40	0.8±0.4
**D298**	8.6±0.3	0.1±0	1.3±0.3	2.5±0.2	2.4±0.8	5.5±0.7	1639.9±568	192.0±44	76.5±10	305.2±60	0.6±0.1
**D328**	8.8±0.2	0.1±0	1.3±0.3	3.2±0.4	2.7±0.8	4.6±0.2	1371.9±138	210.8±9	69.2±3	21.1±7	62.8±15
**D355**	8.7±0.3	0.1±0	1.3±0.2	3.3±0.1	2.2±0.9	5.4±0.3	1478.7±228	202.7±23	75.1±19	131.2±31	2.4±0.4

Supp. Figure 1

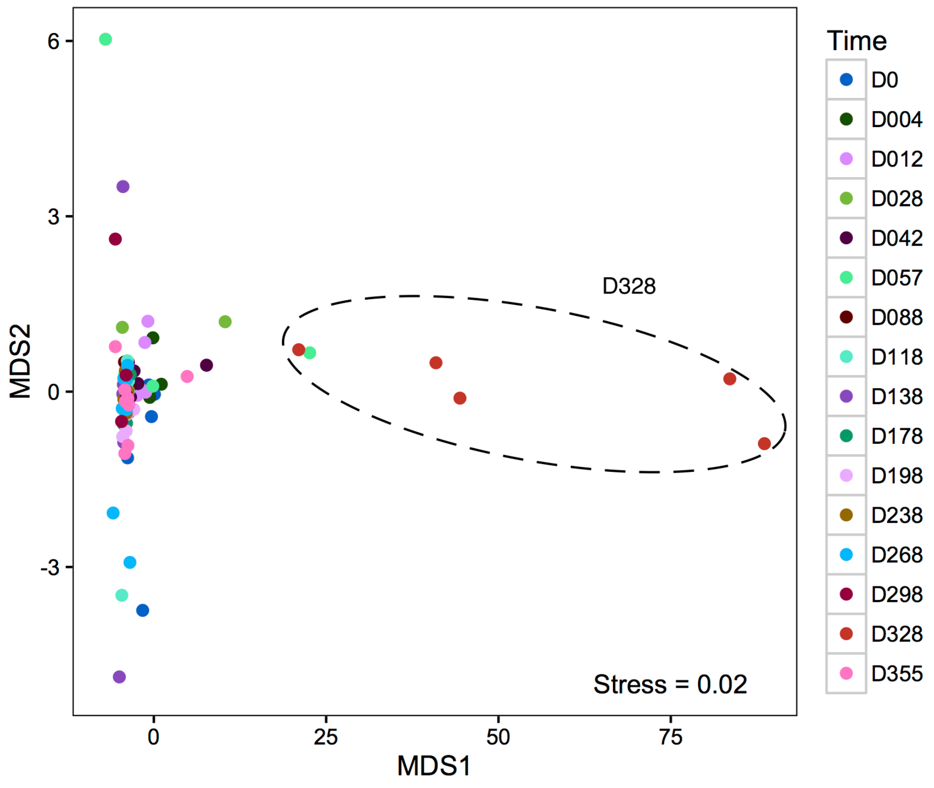

Supp. Figure 2

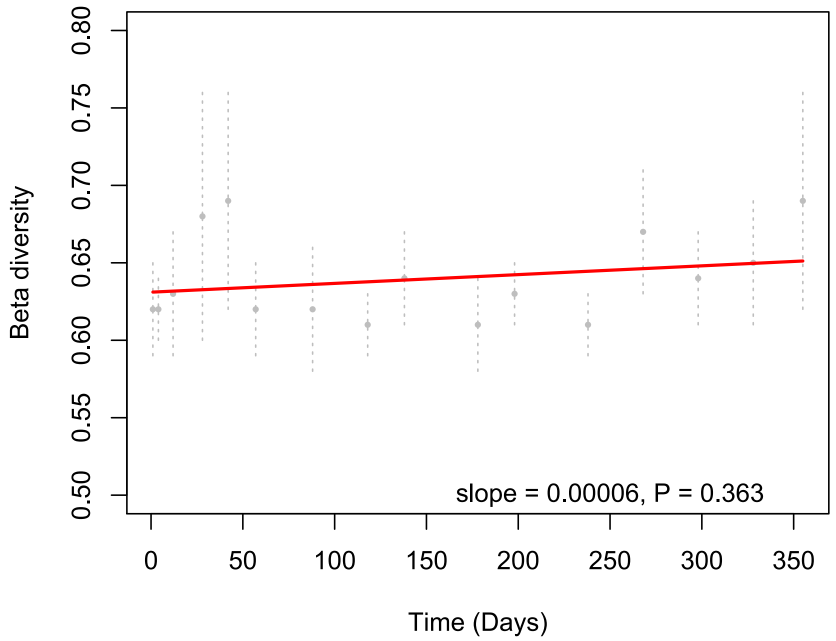

Supp. Figure 3

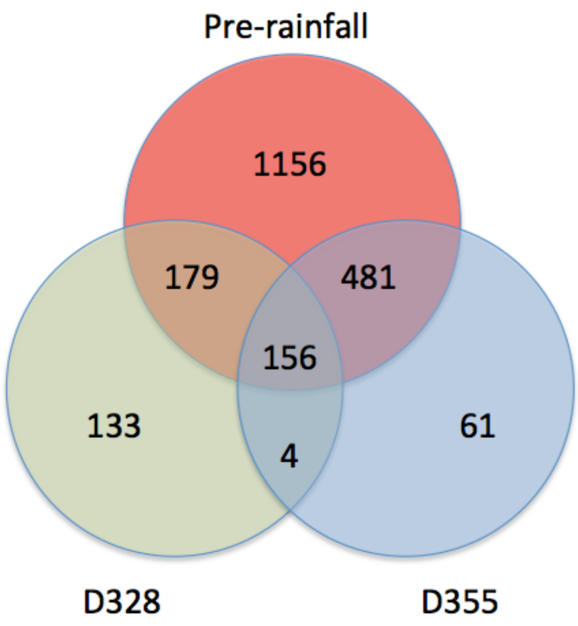

Supp. Figure 4

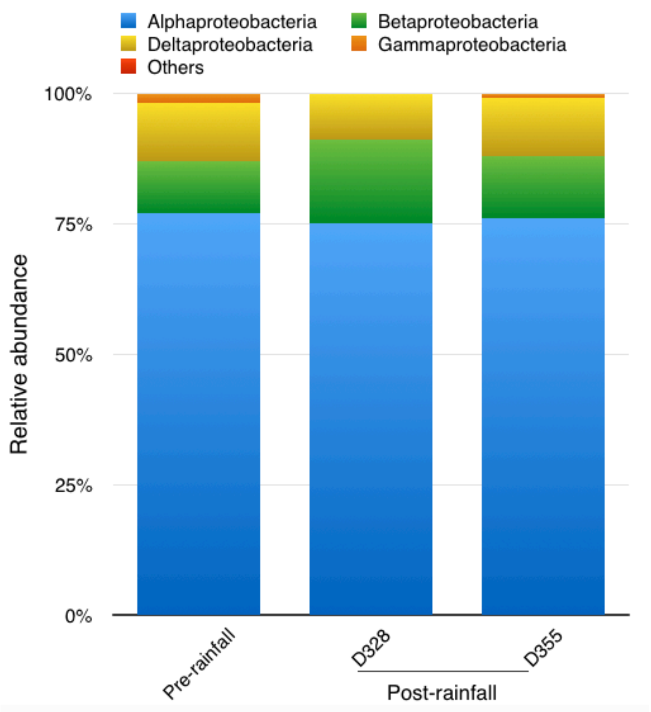

Supplement: Supplementary Information [file srep34434-s1.pdf]
